# Novel application of Ki67 to quantify antigen-specific *in vitro* lymphoproliferation

**DOI:** 10.1016/j.jim.2010.08.007

**Published:** 2010-10-31

**Authors:** Andreia Soares, Lerisa Govender, Jane Hughes, Wendy Mavakla, Marwou de Kock, Charlene Barnard, Bernadette Pienaar, Esme Janse van Rensburg, Gail Jacobs, Gloria Khomba, Lynnette Stone, Brian Abel, Thomas J. Scriba, Willem A. Hanekom

**Affiliations:** South African Tuberculosis Vaccine Initiative, Institute of Infectious Diseases and Molecular Medicine and School of Child and Adolescent Health, University of Cape Town, Cape Town, South Africa

**Keywords:** PPD, purified protein derivative, TT, tetanus toxoid, OG, Oregon Green, Ki67, T cells, Cellular proliferation, Vaccine, Clinical immunology

## Abstract

Antigen-specific proliferation is a critical function of memory T cells that is often utilised to measure vaccine immunogenicity and T cell function. We proposed that measurement of intracellular expression of the nuclear protein, Ki67, could reliably assess specific T cell proliferation *in vitro*.

Ki67 was expressed in CD4+ and CD8+ T cells that had undergone *in vitro* proliferation after 6-day culture of human whole blood or PBMC with antigens. T cells cultured with no antigen did not express Ki67. When compared to current flow cytometry based proliferation assays, Ki67 detected proliferating cells with greater sensitivity than BrdU incorporation, whereas its sensitivity was similar to dye dilution of Oregon Green (OG), a CFSE derivative. Overall, the magnitude and cytokine expression profile of proliferating T cells detected by Ki67 expression correlated strongly with T cells detected with BrdU or OG. The intra-assay variability of Ki67 proliferation was 2–3% for CD4+ T cells, and 10–16% for CD8+ T cells. Finally, we demonstrate that the Ki67 assay detects tetanus toxoid-specific CD4+ T cell proliferation after infant vaccination with tetanus toxoid (TT).

Overall our data suggest that intracellular Ki67 expression provides a specific, quantitative and reproducible measure of antigen-specific T cell proliferation *in vitro*.

## 1 Introduction

Proliferation and clonal expansion of antigen-specific T cells are critical functions for mediating protective immunity and immunological memory (Rosenberg et al., 1997; Combadiere et al., 2004). Previously, the most widely used method for detection of antigen-specific T cell proliferation has involved incorporation of ^3^H-thymidine into DNA of dividing cells (Payan et al., 1983; Marchant et al., 1999). This technique has largely been replaced by flow cytometric assays of proliferation. Examples include fluorescent dye dilution assays, using CFSE or its derivative, Oregon Green (OG) (Magg and Albert 2007; Wallace et al., 2008; MacMillan et al., 2009), and assays that detect the DNA intercalating agent, 5-bromo-2′-deoxyuridine (BrdU), detected by fluorochrome-conjugated antibody staining (Dolbeare et al., 1983; Houck and Loken 1985; Rosato et al., 2001). The advantages of these assays are that they allow co-staining with other markers, enabling delineation of cellular sub-populations according to phenotype and functional characteristics, such as cytokine production (Lyons, 2000; Bachmann et al., 2005; Precopio et al., 2007).

Ki67 is a nuclear protein that plays a role in the regulation of cell division. This marker has been used extensively in cancer biology to indicate tumour cell proliferation (Gerdes, 1990; Scholzen and Gerdes, 2000). The protein is expressed during all active phases of cell division, but is absent in quiescent cells and during DNA repair (Gerdes et al., 1984). Intracellular Ki67 expression directly *ex vivo*, or after *in vitro* cell culture, has been used to measure specific T cell responses induced by vaccination (Stubbe et al., 2006; Cellerai et al., 2007; Miller et al., 2008), or turnover of these cells in individuals with chronic viral infections, such as HIV infection (Sachsenberg et al., 1998; Doisne et al., 2004).

In this study, we show that Ki67 expression in T cells is a specific and quantitative indicator of proliferation, and that results are comparable to those when proliferation is measured by other methods. We also show that measurement of Ki67 may be applied to longitudinal monitoring of vaccine-specific T cell responses. Overall, the Ki67 assay offers a reliable, versatile and simple method for detection of antigen-specific T cell proliferation.

## 2 Materials and methods

### 2.1 Study subjects

Healthy adult donors were recruited at the Institute of Infectious Disease and Molecular Medicine, University of Cape Town. Healthy, 18 month old toddlers were recruited at the South African Tuberculosis Vaccine Initiative clinic sites in the Western Cape, South Africa, before, and 11–13 days after their routine 18 month vaccination with TT. Enrolled toddlers had received all routine childhood vaccinations as set out by the WHO Expanded Programme on Immunisation. Heparinised venous blood from adults and toddlers was collected into BD Vacutainer CPT tubes (BD Biosciences) and immediately processed as outlined below. Participation of all participants was in accordance with the Declaration of Helsinki, the US Department of Health and Human Services guidelines, and good clinical practice guidelines. This included protocol approval by the Research Ethics Committee of the University of Cape Town, and written informed consent by all adults or parents of the toddlers.

### 2.2 Whole blood BrdU incorporation assay

Whole blood (125 μL diluted 1:10 in warm RPMI 1640) was incubated with antigens for 6 days at 37 °C with 5% CO_2_. Antigens were used at the following final concentrations: 1 × 10^5^ cfu/mL Danish BCG (Danish strain 1331; Statens Serum Institut), 1 μg/mL TB10.4 protein (kindly provided by Tom Ottenhoff, Leiden University, Leiden, Netherlands), 2 μg/mL *M. tuberculosis* purified protein derivative (PPD, Statens Serum Institut) and 0.16 IU TT (Tetavax, Sanofi Pasteur). On day 6 (day 3 for PHA), 10 μmol/L BrdU (Sigma-Aldrich) was added for the last 5 h of culture. When intracellular cytokine expression was assessed, 10 ng/mL phorbol 12-myristate 13-acetate (PMA, Sigma-Aldrich), 1.5 μg/mL ionomycin (Sigma-Aldrich) and 1.5 μg/mL Brefeldin A (Sigma-Aldrich) were also added during the last 5 h of culture. Control antigens included 1 μg/mL phytohaemagglutinin (PHA; positive control, Sigma-Aldrich), medium only (unstim., negative control) or, for intracellular cytokine assays, medium with PMA and ionomycin (unstim-PI). On day 6, cells were harvested with 2 mM EDTA (Sigma-Aldrich) and red blood cells lysed. White cells were stained with a viability dye (LIVE/DEAD Fixable Violet Dead Cell Stain Kit, Invitrogen), fixed in BD FACS Lysing Solution (BD Biosciences) according to manufacturer's instructions and cryopreserved until analysis.

### 2.3 PBMC isolation and the OG assay

PBMC were isolated by density gradient centrifugation and immediately stained with 10 μg/mL of CellTrace Oregon Green 488 (Molecular Probes, Invitrogen) per 1 × 10^7^ cells and rested overnight at 37 °C, 5% CO_2_. Cells were either incubated with medium or 1 × 10^5^ cfu/mL Danish BCG, 0.5 μg/mL PPD, 1 μg/mL TB10.4 protein or 0.05 μg/mL staphylococcal enterotoxin B (SEB, positive control, Sigma-Aldrich), for 6 days at 37 °C with 5% CO_2_. On day 6 for some assays, PBMC were restimulated with 50 ng/mL PMA, 250 ng/mL ionomycin and 10 μg/mL Brefeldin A for a further 5 h. Finally, PBMC were stained with LIVE/DEAD Fixable Violet Dead Cell Stain, fixed with BD FACS Lysing Solution (BD Biosciences) and cryopreserved until analysis.

### 2.4 Antibodies and flow cytometry

The following monoclonal antibodies were used for phenotypic and/or intracellular cytokine staining: CD3-QDot 605 (UCHT1), CD4-PerCP (SK3), CD8-PerCP-Cy5.5 (SK1), Ki67-PE (B56), IFN-γ-Alexa Fluor 700 (B27), TNF-α-PE-Cy7 (MAb11), IL-2-APC (MQ1-17H12), and anti-BrdU-FITC (B44). All antibodies were from BD Biosciences except for CD3-QDot 605, which was from Invitrogen. Samples were acquired on a BD LSRII flow cytometer (BD Biosciences, San Jose, CA).

### 2.5 Data analysis

Cell doublets were excluded using forward scatter-area versus forward scatter-height parameters. Single-stained or unstained mouse κ beads were used to calculate compensations for every run. In some experiments CD4+ T cells were gated as CD3+ CD8− lymphocytes, because PMA and ionomycin stimulation strongly down-regulates CD4 expression on T cells. Data were analysed with FlowJo software v.8.8.6 (Treestar Inc.), Pestle v 1.6.2 and Spice v 4.3.2 software (provided by M. Roederer, National Institutes of Health, Bethesda, MD). Statistical analyses were calculated using GraphPad Prism v 4.0.

## 3 Results

### 3.1 Ki67 is a specific marker of *in vitro* lymphoproliferation

**Fig. 1:**
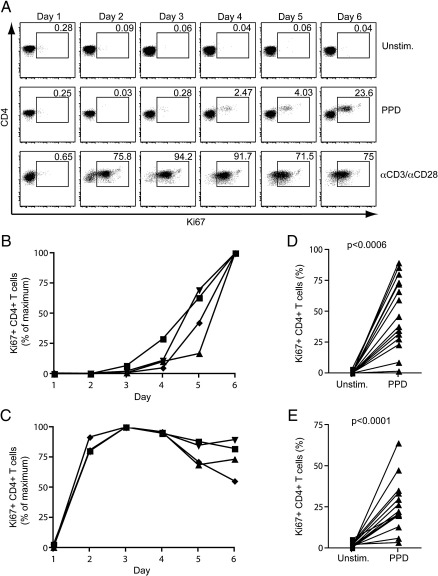
Ki67 as a specific marker of *in vitro* lymphoproliferation. Whole blood from healthy donors was incubated with the indicated antigens and Ki67 expression quantified on a daily basis over 6 days. (A) Representative example showing the frequencies of Ki67 expression by CD4+ T cells after incubation of whole blood with medium only (unstim.), PPD or αCD3/αCD28 over 6 days. Dotplots were gated on live, CD3+ CD4+ lymphocytes. Ki67+ CD4+ T cell frequencies after (B) PPD stimulation or (C) αCD3/αCD28 stimulation in 4 donors. Data are expressed as a percentage of the maximum response. The frequency of Ki67+ CD4+ T cells is indicated in each plot. (D) Frequencies of Ki67 expressing CD4+ T cells in whole blood from 15 donors after 6-day culture with medium only (unstim.) or PPD. (E) Frequencies of Ki67+ CD4+ T cells in PBMC from 14 donors. Differences were calculated using the Wilcoxon matched pairs test.

Ki67 is expressed by all cells undergoing cycling (Lopez et al., 1991; Scholzen and Gerdes, 2000). We investigated the kinetics of Ki67 expression in T cells cultured over 6 days. Whole blood was either cultured in the absence of antigen (unstimulated), or in the presence of purified protein derivative (PPD) or anti-CD3 and anti-CD28 (αCD3/αCD28). Expression of Ki67 was quantified each day. Ki67 expression was low in unstimulated CD4+ T cells on day 1 (24 h, median, 0.62%), and by day 6, had decreased to < 0.1% of CD4+ T cells (median, 0.08%, Fig. 1A). PPD stimulation resulted in Ki67 expression levels above those in unstimulated cells between days 2 and 4; expression peaked on day 6 (Fig. 1A and B). High expression of Ki67 was observed following polyclonal T cell stimulation with αCD3/αCD28; Ki67 was observed responses were high on day 1 already, peaked on day 3, and declined thereafter (Fig. 1A and C).

Next, we assessed proliferation by Ki67 detection in whole blood from 15 healthy donors, after 6-day culture with no antigen, or with PPD. All donors had undetectable or very low frequencies of Ki67+ CD4+ T cells in unstimulated blood (median, 0.07%). PPD stimulation resulted in higher frequencies of Ki67+ CD4+ T cells in all donors (median, 46.1%, Fig. 1D).

We also determined whether proliferation could be detected by assessing Ki67 expression in PBMC. Again, Ki67 expression identified *in vitro* CD4+ T cell proliferation; frequencies of Ki67+ cells after PPD stimulation consistently exceeded those in unstimulated PBMC, at a median of 21.7% (Fig. 1E).

These data suggest that in 6-day PBMC or whole blood culture with antigen, Ki67 expression is up-regulated in T cells undergoing *in vitro* proliferation.

### 3.2 Comparison of Ki67 expression with BrdU and Oregon Green assays

**Fig. 2:**
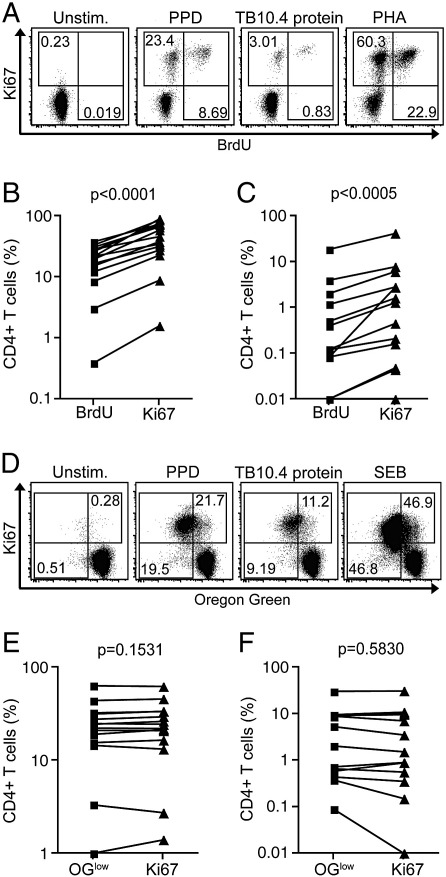
Comparison of the Ki67 proliferation assay with the BrdU and Oregon Green proliferation assays. (A) Representative dotplots showing Ki67 versus BrdU expression by CD4+ T cells in whole blood. Dotplots are gated on live, CD3+ CD8− lymphocytes. Frequencies of (B) PPD- and (C) TB10.4-specific CD4+ T cell proliferation as detected by Ki67 expression or BrdU incorporation (*n* = 15). CD4+ T cells are defined as CD3+ CD8− T cells (see Data analysis). (D) Representative dotplots showing Ki67 and dye dilution of Oregon Green by CD4+ T cells in PBMC. Dotplots are gated on live, CD3+ CD8− lymphocytes. Frequencies of (E) PPD- and (F) TB10.4-specific CD4+ T cell proliferation as detected by Ki67 expression or dye dilution of Oregon Green (OG^low^) in 14 donors. CD4+ T cells are defined as CD3+ CD8− T cells (see Data analysis). Differences were calculated using the Wilcoxon matched pairs test.

Next, we compared our Ki67-based proliferation assay with more traditional flow cytometric proliferation assays, i.e., those measuring BrdU incorporation and dye dilution of OG (Fig. 2).

BrdU is incorporated into cells undergoing DNA synthesis, and is typically added during the last 2 to 24 h of a proliferation assay; in this study we added BrdU for the last 5 h of the 6-day culture. The frequency of Ki67+ CD4+ T cells was higher than the frequency of BrdU+ cells after whole blood stimulation with PPD or TB10.4 protein (Fig. 2A, B and C). Importantly, all BrdU+ cells co-expressed Ki67 (Fig. 2A).

The OG assay requires uniform labelling of cells prior to long-term culture. In contrast to results from the BrdU assay, the OG and Ki67 assays yielded remarkably similar frequencies of proliferating, specific T cells; Ki67+ and OG^low^ CD4+ T cell frequencies were not different in PPD or TB10.4-stimulated PBMC (Fig. 2D, E and F).

**Fig. 3:**
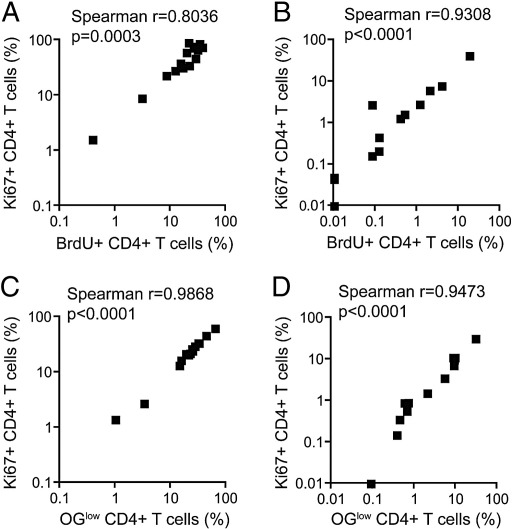
Correlations between Ki67+ CD4+ T cell expression and BrdU incorporation or dye dilution of Oregon Green (OG^low^). Whole blood was incubated with (A) PPD or (C) TB10.4 for 6 days (*n* = 15). PBMC were incubated with (B) PPD or (D) TB10.4 for 6 days (*n* = 14). Correlations were calculated using a Spearman's rank correlation coefficient.

Frequencies of Ki67+ CD4+ T cells correlated strongly with BrdU+ CD4+ T cell frequencies (Fig. 3A and B). Similarly, a strong correlation was found between frequencies of antigen-specific Ki67+ and OG^low^ CD4+ T cells (Fig. 3C and D).

These data show that frequencies of proliferating T cells detected by Ki67 expression agree with frequencies detected with conventional proliferation assays.

### 3.3 Cytokine expression profiles of proliferating CD4+ T cells

**Fig. 4:**
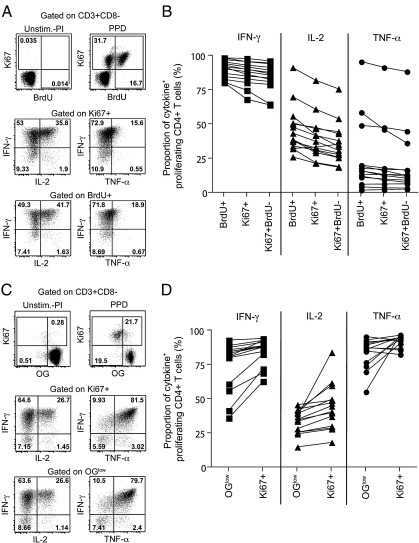
Cytokine expression profiles of proliferating CD4+ T cells. Whole blood or PBMC were cultured for 6 days with no antigen or PPD. On day 6, cells were restimulated with PMA and ionomycin for 4 h in the presence of Brefeldin A to detect cytokine expression by proliferating T cells. Representative dotplots of the cytokine expression profiles of (A) Ki67+ or BrdU+ CD4+ T cells and (C) Ki67+ or OG^low^ CD4+ T cells. (B) Proportions of BrdU+, Ki67+ or Ki67+ BrdU− CD4+ T cells expressing IFN-γ, IL-2 or TNF-α (*n* = 15). (D) Proportions of Ki67+ or OG^low^ CD4+ T cells expressing IFN-γ, IL-2 or TNF-α (*n* = 14).

The functional capacity of cells that have expanded during the 6-day culture may be assessed by short-term polyclonal re-stimulation with PMA and ionomycin on day 6. This induces cytokine production, which can be measured by intracellular staining. We compared expression of IFN-γ, IL-2 and TNF-α by Ki67+ CD4+ T cells with expression of these cytokines in BrdU+ or OG^low^ CD4+ T cells. When Ki67 and BrdU assay results were compared, similar expression of IFN-γ and TNF-α was observed in proliferating CD4+ T cells. BrdU+ CD4+ T cells yielded higher proportions of IL-2+ cells than Ki67+ CD4+ T cells but these differences were small (Fig. 4A and B). Similar expression profiles of IFN-γ, IL-2 and TNF-α were observed when comparing Ki67+ and OG dilution (Fig. 4C and D).

### 3.4 Intra-assay variability of Ki67 proliferation assay

To test the reproducibility of the Ki67 proliferation assay, we performed 5 proliferation assays per donor on whole blood from 3 healthy adult volunteers. Intra-assay coefficient of variation (CV) values for PPD-specific Ki67+ CD4+ T cells were between 2% and 3%, and for Ki67+ CD8+ T cells, which were present at lower frequencies than Ki67+ CD4+ T cells, between 10 and 16%. Even lower CV values were observed for PHA-stimulated blood, which induced the highest frequencies of Ki67+ T cells (Table 1). These results indicate that the Ki67 proliferation assay generates highly reproducible findings.

### 3.5 Measurement of vaccination-induced T cell proliferation

To establish if Ki67 can be used to measure vaccine-specific T cell proliferation, we determined Ki67 expression in T cells before and 11–13 days after tetanus toxoid (TT) re-immunisation of healthy, 18 month old infants. This post-vaccination time point was selected because it coincides with the peak TT-specific CD4+ T cell response in healthy adults (Cellerai et al., 2007).

**Fig. 5:**
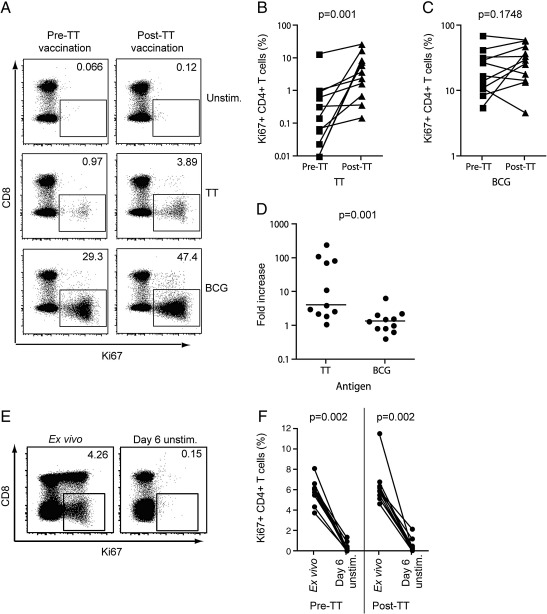
Monitoring of vaccine-induced T cell proliferation. (A) Dotplots showing Ki67 expression by CD4+ T cells from a representative 18 month old toddler before (pre-TT) and after TT vaccination (post-TT). Dotplots are gated on live, CD3+ lymphocytes. Values in each dotplot represent the frequency of Ki67+ T cells within the CD3+ CD8− T cell population. Frequencies of (B) TT-specific and (C) BCG-specific CD4+ T cells pre- and post-TT vaccination in 11 toddlers. CD4+ T cells are defined as CD3+ CD8− T cells (see Data analysis). (D) Relative increase in TT-specific or BCG-specific CD4+ T cells pre- and post-TT. The lines represent the medians. (E) Dotplots depicting frequencies of Ki67+ CD4+ T cells in whole blood directly *ex vivo* or after culture in the absence of antigen (unstim.) for 6 days. Values in each dotplot represent the frequency of Ki67+ T cells within the CD3+ CD8− T cell population. (F) Frequencies of Ki67+ CD4+ T cells directly *ex vivo* or after culture for 6 days with medium (*n* = 11). CD4+ T cells are defined as CD3+ CD8− T cells (see Data analysis). Differences were calculated using the Wilcoxon matched pairs test.

The frequency of proliferating, Ki67+ CD4+ T cells observed pre-vaccination, following *in vitro* incubation of whole blood with TT, was low (median, 0.15%). After vaccination, TT-specific CD4+ T cell proliferation increased markedly (median, 3.77%, Fig. 5A and B). To control for possible non-specific up-regulation of Ki67 after TT vaccination *in vitro*, we also quantified BCG-specific T cell proliferation pre- and post-vaccination. Frequencies of BCG-specific Ki67+ CD4+ T cells were not different before and after TT vaccination (Fig. 5A, C and D).

It is well established that vaccination-induced T cell proliferation results in increased *in vivo* and, thus, *ex vivo* expression of Ki67 (Cellerai et al., 2007; Miller et al., 2008). To determine whether “background” expression levels of Ki67, reflecting *in vivo* T cell turnover, affected the detection of antigen-specific T cell proliferation *in vitro*, we quantified Ki67 expression directly *ex vivo* in whole blood from toddlers before and 11–13 days after TT vaccination. High *ex vivo* frequencies of Ki67+ CD4+ T cells were readily detected in all toddlers before and after TT vaccination (Fig. 5E and F). Importantly, after 6 days of culture in the absence of antigen, Ki67 expression decreased markedly to background levels (Fig. 5E and F).

These data suggest that *in vivo* T cell turnover does not interfere with the specificity of the Ki67 proliferation assay. This assay is therefore specific for the detection of antigen-specific T cell proliferation *in vitro*.

## 4 Discussion

Proliferation is a commonly measured indicator of T cell function. We assessed intracellular Ki67 expression as a marker of *in vitro* proliferation in whole blood or PBMC-based assays. We show that the Ki67 assay provides an alternative approach to measuring antigen-driven T cell proliferation, and found that results obtained were very similar to those generated by commonly used proliferation assay systems.

The development of fluorescent dyes and tracking markers has enabled combined analysis of antigen-specific T cell proliferation, phenotyping and cytokine expression by flow cytometry (Johannisson and Festin, 1995; Mehta and Maino, 1997; Lyons and Doherty, 2004; Wallace et al., 2008). To date, whole blood BrdU and PBMC dye dilution assays have been the preferred flow cytometry based methods to assess lymphocyte proliferation. In comparison, Ki67 expression identified approximately double the frequency of proliferating CD4+ T cells detected by BrdU incorporation. Incubation of cells with BrdU is limited to 24 h or less because incorporated BrdU inhibits cell cycle progression. Therefore a major limitation of the BrdU assay is that only cells that have progressed through the S-phase during this short incubation period may be detected. In contrast, cells express Ki67 in all active phases of the cell cycle. Therefore, Ki67 appears to be a more sensitive marker for the detection of rare T cell responses, and may reflect the extent of *in vitro* antigen-specific proliferation more accurately than BrdU incorporation.

Cellular proliferation in PBMC samples is routinely evaluated by dye dilution methods, using CFSE or derivatives such as OG (Robinson and Amara, 2005). A recent non-human primate study has proposed measurement of *in vitro* proliferation by the combined analysis of Ki67 and side scatter properties of cells (Shedlock et al., 2010). The authors demonstrate a correlation between this assay and the CFSE dilution assay. In this study, we show that the proliferation events detected by loss of OG dye are virtually identical to the Ki67+ events. From this we reasoned that Ki67 expression is an accurate measure of T cell proliferation as only cells that have completed cycling display a decrease in OG fluorescence intensity. Limitations of many protein reactive dye compounds include cellular toxicity (Last'ovicka et al., 2009; Shedlock et al., 2010) and sensitivity to pH and light (Wallace et al., 2008). The Ki67 proliferation assay requires no incubation or washing steps prior to or during the culture, and exposure of cells to toxic compounds is eliminated. Additionally, since labelling of cells is not required before antigen stimulation, detection of Ki67 by flow cytometry can be performed on antigen-stimulated cells after cryopreservation. A limitation of Ki67 as a proliferation marker is its inability to resolve the number of proliferation cycles that cells have undergone, as can be done with dye dilution assays (Parish, 1999; Lyons and Doherty, 2004). Enumeration of cell cycles enables calculation of the original precursor frequency of specific cells, since the number of cells and their respective number of divisions are known (Givan et al., 1999).

Monitoring vaccine-induced T cell proliferative potential is important for determining vaccine take, memory function and long-term persistence of vaccine-specific responses. Previous studies have quantified Ki67 expression directly *ex vivo* as a measure of the vaccine-induced proliferative response (Miller et al., 2008), or in combination with activation markers to identify antigen-specific T cells (Stubbe et al., 2006). To detect increases in the expression of Ki67, these studies relied on low-level Ki67 expression before vaccination in healthy adults. Direct *ex vivo* detection of antigen-specific Ki67 expression may thus be challenging in individuals with high levels of *in vivo* T cell proliferation — such as those resulting from recent vaccinations or infections. We observed high *ex vivo* frequencies of Ki67+ CD4+ T cells in toddlers, suggesting elevated levels of *in vivo* T cell turnover. This turnover is likely to be driven by routine childhood vaccinations and exposure to infections, common in this age group. Whole blood cultured in the absence of antigen reduced Ki67 expression to barely detectable levels by day 6, presumably due to cells reverting to a quiescent state. Therefore, this 6-day assay proved to be sufficiently specific and sensitive for the identification of rare, antigen-specific T cells following vaccination in the context of high *ex vivo* frequencies of Ki67+ T cells.

Overall, our data show that outcomes of the Ki67 assay correlate strongly with current flow cytometry based whole blood and PBMC proliferation assays. This assay is highly reproducible, versatile, and presents several practical advantages over current techniques. We propose Ki67 as a marker for quantifying antigen-specific T cell proliferation, and utilising this assay to monitor T cell responses in large field studies or paediatric studies based on limited blood volumes.

## Conflict of interest

The authors declare no financial or commercial conflicts of interest.

## Figures and Tables

**Table 1:** Intra-assay CV values for T cell frequencies of Ki67 expression after PPD or PHA stimulation.

Subset	Donor 1		Donor 2		Donor 3	
	Ki67+ CD4+	Ki67+ CD8+	Ki67+ CD4+	Ki67+ CD8+	Ki67+ CD4+	Ki67+ CD8+
*PPD stimulation*						
Mean	66.2	10.31	79.82	8.3	62.79	3.03
SD	1.88	1.66	1.46	0.86	1.33	0.34
CV	2.84	16.14	1.83	10.42	2.12	11.17

*PHA stimulation*						
Mean	94.5	91.26	94.7	91.7	76.65	74.54
SD	0.87	1.14	0.81	1.11	3.32	2.4
CV	0.92	1.25	0.86	1.21	4.33	3.21
